# Seroprevalence of *Mycoplasma gallisepticum* antibody by ELISA and serum plate agglutination test of laying chicken

**DOI:** 10.14202/vetworld.2015.9-14

**Published:** 2015-01-02

**Authors:** Md. Zulfekar Ali, Md. Mostafizer Rahman, Shirin Sultana

**Affiliations:** 1 Department of Microbiology, Hajee Mohammad Danesh Science and Technology University, Dinajpur, Bangladesh; 2 Department of Pathology and Parasitology, Hajee Mohammad Danesh Science and Technology University, Dinajpur, Bangladesh

**Keywords:** antibodies, *Mycoplasma gallisepticum*, layer chickens, seroprevalence

## Abstract

**Aim:**

*Mycoplasma gallisepticum* (MG) is important avian pathogen responsible for chronic respiratory disease of chicken and turkeys, which result in large economic loss for the poultry industry. The objectives of this study were determination of seroprevalence of MG antibody of commercial layer chicken at laying period in selected areas of Bangladesh.

**Materials and Methods:**

A total of 563 blood samples were collected randomly from selected commercial layer chickens at laying period during the period from July to December, 2013. Indirect enzyme linked immunosorbent assay (iELISA) and serum plate agglutination (SPA) test were performed to detect the presence of antibodies against MG.

**Results:**

Of 563 samples, 64.47% and 56.13% showed an overall prevalence of MG antibodies in iELISA and SPA test respectively. Prevalence of MG was recorded the highest (69.63%) at 50-55 weeks of age compared with lowest (53.26%) at 56-61 weeks of age (p<0.05). Significant (p<0.05) effect of breed were observed in the seroprevalence of MG infection in layer birds in the present study. The overall, 68.77%, 63.74% and 59.37% prevalence were found respectively in sonali, ISA Brown and White leg horn. The prevalence of MG antibodies was the highest (70.13%) in December followed by November (68%), October (65.67%), August (63.46%), September (58.54%) and July (51.78%) month. The seroprevalence of MG antibodies was higher (69.63%) in most of the large flocks and lower (56.82%) in small flocks.

**Conclusion:**

Therefore, might be suggested that the commercial layer farms should be routinely checked to monitor MG infection and the reactor birds should be culled since MG organism has the potential to transmit vertically. The correlation between MG antibody in month and flock size was not significant (p=0.359 and p=0.868, respectively).

## Introduction

Poultry rearing is playing a role for the poverty alleviation and income generation in Bangladesh. At present, there are more than 130 hatcheries producing 3.4 million day old chicks per week and about 30,000 commercial broiler and layer farms supplying 0.2 million metric tons of poultry meat and 5210 million table eggs per year [1]. A total of 5 million people is working presently in this sector and amount of Tk. 1,22,000 million has been invested [1].

One of the major constraints of poultry farming in Bangladesh is the outbreaks of different infectious diseases. *Mycoplasma gallisepticum* (MG) is a highly infectious respiratory pathogen affecting poultry. In breeders and layers, the disease causes a drop in egg production and an increase in embryo mortality [2]. Production losses between 10 and 20% have been reported in layers [3]. All ages of chickens and turkeys are susceptible to these diseases but young birds are more prone to infection than adults [4]. In Bangladesh the prevalence of mycoplasmosis markedly increased in the winter season and may reach up to 61.45% that is a threat for our poultry sector [5].

Like other poultry producing countries, Mycoplasmosis is one of the important disease problems for poultry in Bangladesh, both for commercial exotic breeds and indigenous local breeds. Seroprevalence study of MG in pure breed, hybrid and commercial chickens of six divisions of Bangladesh were conducted by [6]. The overall prevalence was 57.15%. The highest prevalence (77%) was found in Kasila, a broiler hybrid; followed by 76.92% in Lohman brown, 73.19% in Starbrow, 72% in Fayomi, 70% in Isabrown, 65.56% in White leghorn, and 39.28% in Babcock. A survey of commercial egg laying poultry in United States of America (USA) revealed that 37% of laying flocks (262.6 million layers) were infected with MG and causing an annual losses of 97 million US $ [7]. In addition, medication costs make this disease one of the costliest disease problems confronting the poultry industry and causes problems in food safety, drug resistance, and drug residual [2].

It is difficult to diagnose MG infections in poultry flocks based on clinical signs. Routine culture procedures and serology are commonly used. The diagnosis of MG infection traditionally has been done by serology [3]. Several serological tests have been used to detect MG antibodies, but specificity and sensitivity have been lacking to some degree in all of them. They are more satisfactory for flock screening than for testing individual birds. The most commonly used are the serum plate agglutination (SPA) test, the enzyme linked immunosorbent assay (ELISA) and the hemagglutination inhibition (HI) tests. In the SPA test, sera from individual birds are tested for agglutination using commercially produced stained MG antigen [8].

## Materials and Methods

### Ethical approval

This study was conducted after approval by research committee and Institutional animal ethics committee.

### Study area and chicken population

The study was conducted on 28,150 commercial layer chickens at laying period of 12 farms which included four Sonali layer, four ISA Brown and four White leghorn chicken breeds in five upazilas (Sadar upazila, Sariakandi upazila, Gabtoli upazila and Sherpur upazila) of Bogra District during the period from July to December, 2013.

### Experimental layout

Selection of 12 layer farms at different area of Bogra district and collection of blood samples from selected laying birds at laying period then transport the blood samples to laboratory by using ice box. Serum separation was done and performed two serological tests indirect ELISA MG and SPA for MG antibody.

### Collection and preparation of samples

A total of 563 (2% birds of total population) laying hens of 12 farms were selected randomly for serological test. The studied commercial layer had not been previously vaccinated. Blood samples were collected aseptically from wing vein of individual birds with 3 ml sterilized disposable plastic syringe without anticoagulant and allowed to clot for 1 h in the syringe. Blood containing syringe were kept in the refrigerator at 4°C for 4-5 h. The serum (liquid portion) was decanted in centrifuge tube and centrifuge at 2,500 rpm for 5 min to have clear serum. The serum was then collected in sterile eppendorf tube and then preserved at -20° C until further processing for the serological study.

### Detection of specific antibody by indirect ELISA test

**Figure-1:**
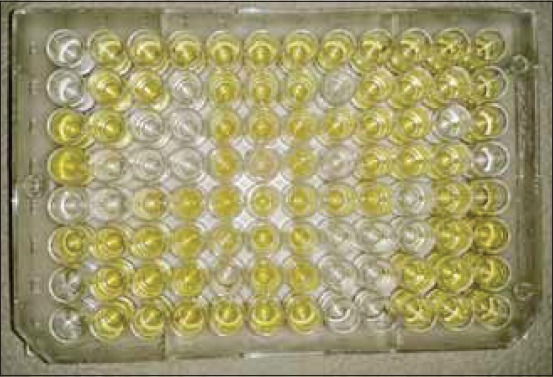
Yellow colour develops in positive case.

MG coated plate (BioChek MG ELISA Kit, Lot No. CK114, Manufactured by BioChek UK Ltd.Co. TW4 5PY Hounslow, UK) was removed from sealed bag and record location of samples on template. The 100 ^l of negative control was added into wells A1 and B1 and 100 ^l of positive control was added into wells C1 and D1. Then 100 ^l of diluted samples were added into the appropriate wells. Cover plate with lid and incubated at room temperature (22-27°C) for 30 min. Contents of wells were aspirated and washed with 4 times by buffer (350 ^l per well). Invert plate and tap firmly on absorbent paper until no moisture were visible. 100 ^l of Conjugate reagent was added into the appropriate wells. Cover plate with lid and incubated at room temperature (22-27°C) for 30 min. Then plate was washed with 4 times by buffer (350 ^l per well). A volume of 100 ^l of Substrate reagent was added into the appropriate wells. Cover plate with lid and incubated at room temperature (22-27°C) for 15 min and observe the colorless wells to yellowish color in positive case (Figure-1). 100 ^l of Stop solution was added to appropriate wells to stop reaction. Finally, the plate placed on ELISA Reader and recorded the absorbance of controls and the samples by reading at 405 nm.

### Calculation of results

The presence or absence of antibody to MG is determined by relating the value of unknown to the positive control mean. The positive control has been standardized and represents significant antibody levels to MG in chicken serum. The relative level of antibody in the unknown can be determined by calculating the sample to positive (S/P) ratio. The equation of calculation provided in ELISA kit was used for the calculation of antibody titer.

a).Negative control mean (NCX): NCX (OD value of well A1 + OD value of well B1) 3 2.
b).Positive control mean (PCX): PCX = (OD value of well C1 + OD value of well D1) 3 2.
c).S/P ratio: S/P= (Mean of test sample - Mean of negative control) 3 (Mean of positive control - Mean of negative control).
d).Antibody titer: The following equation relates the S/P of the sample at a 1:500 dilution to an end point titer-Log_10_ Titer = 1.1 (Log_10_ S/P) + 3.156.

### Interpretation of results

A serum samples with S/P ratios of <0.5 considered negative. S/P ratios >0.5 considered positive and indicates vaccination or other exposure to MG.

### SPA test

**Figure-2:**
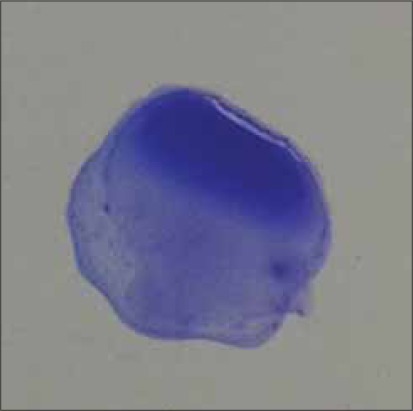
Small clumps, no background clearing (+) by SPA test.

**Figure-3:**
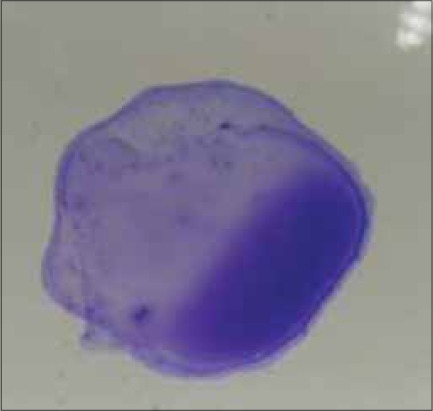
Medium sized clumps almost complete background clearing (++) by SPA test.

**Figure-4:**
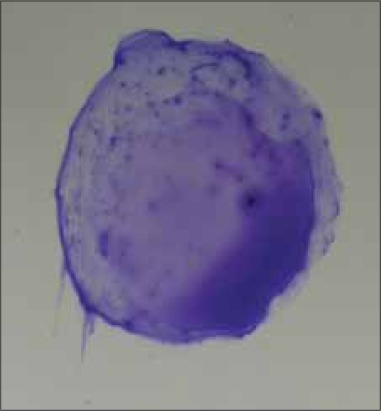
Large clumps, almost complete background clearing (+++) by SPA test.

**Figure-5:**
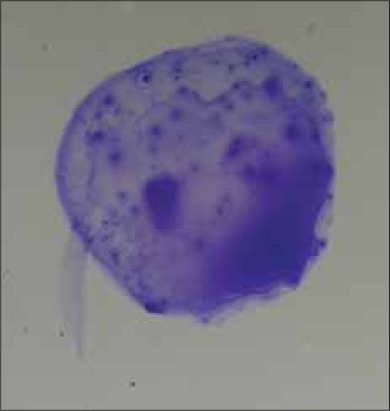
Very large clumps, mostly in the periphery, complete background clearing (++++) by SPA test.

The SPA test was conducted according to the instructions of OIE Manual (2008) [8]. For this test 0.02 ml of MG antigen (Lilli test MG RSA Antigen,

Manufactured by Lillidale Diagnostics, Pig Oak Farm, Holt, Wimborne, Dorset) and 0.02 ml of chicken serum were placed side by side with micropipettes on a glass plate. Then antigen and serum sample were mixed thoroughly by stirring with a small tooth pick. The glass plate was illuminated from below so as to facilitate observing the reaction, avoiding excessive heat from the light source. Positive reaction was characterized by the formation of definite clumps within 2 min after mixing the test serum with antigen (Figure-2 to 5). The clumps usually started appearing and became concentrated at the periphery of the mixture. Negative reaction was judged by the absence of agglutination reaction. Care was taken so that the natural granulation of the antigen showed not to be taken as a positive reaction.

### Statistical analysis

All the data obtained during the study were analyzed statistically to find out the P-value. The P-value and the values in average were determined by ANOVA.

## Results

The seroprevalence of MG antibody in commercial layer farms at laying period of different upazila of Bogra district was studied by indirect ELISA (iELISA) test and SPA test. A total of 563 sera sample were collected and subjected to iELISA and SPA test, from which 363 and 316 sera samples were given positive result by iELISA and SPA test respectively that is showing specific antibody (Ig G) against MG. The prevalence ofMG antibody in the farm Sapla Poultry and Hatchery Ltd. was 71.43% and 63.09%, Northern Poultry Ltd. was 53.33% and 46.44%, Green Poultry Ltd. was 55.55% and 44.44%, Mondol Poultry was 64.61% and 54.41%, Khondokar Poultry was 68.57% and 60%, Haque Poultry was 60.86% and 52.17%, Nahid Poultry was 67.50% and 60%, Bondhu Poultry was 68.33% and 60%, Srabonee Poultry was 65.71% and 57.14%, Aslam Poultry was 65.62% and 56.25%, Rita Poultry was 62.00% and 54% and Mohasthan Poultry 50.00% and 42.31%. The highest prevalence of MG antibody was found to be 71.43% by iELISA and 63.09% by SPA test in Sapla Poultry and Hatchery Ltd. and overall prevalence found 64.47% and 56.13% by iELISA and SPA test respectively. However, the overall results of prevalence of MG antibody shown in Table-1, depicts details of12 farms from where blood samples were collected.

**Table 1:** Seroprevalence of MG antibody by iELISA and SPA test.

Name of farm	Age (wks)	Number of sera tested	iELISA			SPA	Overall prevalence	
		
			No. of positive samples and %	Mean titer	CV%	No. of positive samples and %	iELISA	SPA
Sapla Poultry and Hatchery Ltd.	55	84	60 (71.43)	787	45	53 (63.09)	64.47%	56.13%
Northern Poultry Ltd.	59	30	16 (53.33)	666	67	14 (46.44)		
Green Poultry Ltd.	61	36	20 (55.55)	647	68	16 (44.44)		
Mondol Poultry	46	54	35 (64.61)	720	55	31 (54.41)		
Khondokar Poultry	50	70	48 (68.57)	690	64	42 (60)		
Haque Poultry	49	46	28 (60.86)	708	60	24 (52.17)		
Nahid Poultry	38	40	27 (67.50)	725	58	24 (60)		
Bondhu Poultry	53	60	41 (68.33)	744	62	36 (60)		
Srabonee Poultry	43	35	23 (65.71)	695	67	20 (57.14)		
Aslam Poultry	40	32	21 (65.62)	698	64	18 (56.25)		
Rita Poultry	44	50	31 (62.00)	719	49	27 (54)		
Mohasthan Poultry	56	26	13 (50.00)	528	98	11 (42.31)		
Total=		563	364 (64.47)			316 (56.13)		

iELISA=Indirect enzyme linked immunosorbent assay, CV%= Coefficient of variance %, SPA=Serum plate agglutination, MG=*Mycoplasma gallisepticum*

From the Table-2, it is evident that the prevalence of MG antibody was the highest in commercial layer of age 50-55 weeks that was 69.69% and the 2^nd^ highest prevalence was found for the commercial layer of age 38-43 weeks. The lowest prevalence of MG antibodies was found at 56-61 weeks of age.

**Table-2:** Seroprevalence of MG antibody by age.

Age of birds (Weeks)	Number of sera tested	Number of positive samples	Prevalence %	P value
38-43	107	71	66.35	0.068*
44-49	150	94	62.66	
50-55	214	149	69.63	
56-61	92	49	53.26	

* *Significant (p<0.05),* MG=*Mycoplasma gallisepticum*

The prevalence of MG antibodies among three commercial layer breeds Sonali was shown higher that was 68.77% and lower prevalence was white leg horn that was 59.37% (details in Table-3).

**Table-3:** Seroprevalence of MG antibody in different breeds of poultry.

Name of Breeds	Number of sera tested	Number of positive samples	Prevalence %	P value
Sonali	221	152	68.77	0.027*
ISA Brown	182	116	63.74	
White leg horn	160	95	59.37	

* *Significant (p<0.05),* MG=*Mycoplasma gallisepticum*

The prevalence of MG antibodies was higher in December month that was 70.13% among 6 months during study period and the 2^nd^ highest prevalence was found for the month of November that was 68%. Other 4 months was shown 51.78%, 63.46%, 58.54% and 65.67% were July, August, September and October month respectively (Table-4).

**Table-4:** Seroprevalence of MG antibody by month.

Name of month	Number of sera tested	Number of positive samples	Prevalence %	P value
July	56	29	51.78	0.359^NS^
August	104	66	63.46	
September	82	48	58.54	
October	67	44	65.67	
November	100	68	68.00	
December	154	108	70.13	

*NS=Not significant (p>0.05),* MG=*Mycoplasma gallisepticum*

The prevalence of MG antibody of commercial layer chicken at laying stage according to flock size was higher in larger flock size. The highest prevalence was shown in flock size 3000-4200 that was 69.63% and 2^nd^ highest prevalence was shown in flock size 1700-2000 that was 63.06% and lowest prevalence was shown in flock size 1300-1600 that was 56.82%. The details of the result of relation to flock size are shown in Table-5.

**Table-5:** Seroprevalence of MG antibody in relation to flock size.

Flock size (No. of birds/flock)	Number of sera tested	Number of positive samples	Prevalence	P value %
1300-1600	88	50	56.82	0.868^NS^
1700-2000	111	70	63.06	
2300-2700	150	94	62.66	
3000-4200	214	149	69.63	

*NS=Not significant (p>0.05),* MG=*Mycoplasma gallisepticum*

## Discussion

A total of 563 sera samples were collected from 12 commercial layer farms at laying period and were subjected to iELISA and SPA test. Out of these, 363 (64.47%) sample were found positive for MG infection by iELISA test (Table-2). The overall prevalence of MG infection was 64.47%. Similar reports have been described by [9] who detect the status of IgG antibodies of MG by iELISA test on broiler breeding stocks as highest (74.60%) in Lahore district of Pakistan and another researcher [10] recorded 33.3% in northwest of Iran. Another author [11] recorded 65.2% seroprevalence of MG antibodies on commercial layer chickens in the laying period that was more similar to my findings. For comparison purpose, a number of studies on laying hens from different countries for the prevalence of MG by iELISA test that is agreement with previous reports of [12] in

France, [13] in Italy, [14] in Egypt and [15] in Jordan who reported 84%, 31%, 60% and 73.5% respectively. On the other hand the same samples were tested by SPA test and found 56.13% overall seroprevalence that was 8.35% lower from tested with iELISA test (Table-1). Similar reports have been described by [5] who reported 56.9% seropositive layer chickens tested with SPA for MG infection in Potuakhali district of Bangladesh. Another researcher [16] who demonstrates 58.9% prevalence of MG in layer chickens in some model breeder poultry farms in Feni district of Bangladesh. About 45.1% seroprevalence of MG was carried out in layer chickens in Rajshahi and surrounding districts of Bangladesh [17]. My finding also is in agreement with previous reports of [18-20] and who reported 56.5%, 59.1% and 53.0% seroprevalence of MG infection in chickens, respectively. The overall prevalence of MG infection was recorded as 67.4% on 96 commercial layer farms of six upazilas of Khulna district [21].

Age-wise analysis revealed that the highest prevalence of MG infection was 69.63% in 50-55 weeks age group followed by 66.35% in 38-43 weeks age group. The lowest prevalence was 53.26% in age group of 56-61 weeks (Table-2). Statistically significant (P<0.05) difference was seen in bird age and MG prevalence. Similar observation was made by [17] who recorded the highest prevalence (71.6%) at 16-23 weeks of age and lowest (50.4%) at >64 weeks of age. Some other earlier reports also support the present finding where lowest prevalence was recorded in birds of >55 weeks of age and highest at 18-20 weeks of age [16]; [5]. Another researcher [9] was recorded highest (74.60%) of MG infection was found in aging from 6 to 23 weeks while lowest (33.17%) in flocks of 60-76^th^ weeks of age.

The prevalence varied widely among different breeds of chickens. These differences might have happened due to breed variation, nature of poultry farming, operational practices and other biosecurity measures of the farms [22]; [6] and [23]. The highest prevalence was found in Sonali breeds that were 68.77%, followed by 63.74% in ISA Brown and lowest prevalence (59.37%) was found in White leg horn. Significant (P>0.05) difference was seen in breed and MG prevalence. Similar observation demonstrated by [23] she recorded the overall prevalence of MG was 52% in Sonali breed and [6] observations on White leghorn was 39.29% that is lower than 59.37% from my findings.

The highest prevalence of MG infection was found in November and December that is 68.00% and 70.13% respectively due to low temperature and lowest prevalence was found during July and August that is 51.78% and 63.46% respectively due to hot temperature. Not significant (P>0.05) difference was seen in month and MG prevalence. It was found that cold weather and high relative humidity can influence the prevalence. Similar report was demonstrated by [17] who reported 61.6% prevalence of MG in winter followed by autumn 56.9%, rainy 55.0% and summer 49.7%. My findings also supported the report of [5] who recorded highest prevalence in winter season (61.45%) than the rainy season (51.74%). In Algeria [24] recorded MG infection significantly higher during winter season (61.48%) than in summer (47.74%). This finding is in accordance with the reports of earlier observers who reported higher prevalence in winter season in comparison to summer and rainy seasons [21]; [16] and [25]. MG infection more prevalent in winter season (45.13%) in comparison with the summer season (36.30%) in layer flocks in district Faisalabad of Pakistan was recorded by [26].

Serological investigation was shown highest infection rate (69.63%) in large scale flocks (30004200 birds) compared to small (1300-1600 birds) flocks (Table-5) which is strongly supported by the previous investigations of [24] which recorded 76.97% for MG infection in a herd containing 18000 birds from 20% in a herd with 500-1000 birds in Algeria, [17] reported MG infection rate was the highest (68.5%) in large flocks compared to small flocks (50.1%) in Rajshahi and Surrounding Districts of Bangladesh. No significant (P>0.05) difference was seen in flock size and MG prevalence. Similar report also demonstrated by [21] which recorded 90.70% prevalence in larger flock and 63.25% in smaller flock and [26] recorded 48.11% and 27.27 % in high bird density and low bird density respectively.

## Authors’ Contributions

The present study is a thesis part of MS degree of MZA. MMR designed the study and MZA done the research under the guidance of MMR. SS guided and contributed in sample collection and statistical analysis. All authors participated in draft and revision of the manuscript. All authors read and approved the final manuscript.
